# Therapeutic efficacy of SipD/LptD-specific IgY entrapped in alginate nanoparticles against *Salmonella* Typhimurium infection

**DOI:** 10.1016/j.heliyon.2024.e39650

**Published:** 2024-10-24

**Authors:** Mojtaba zafarmand-samarin, Shahram Nazarian, Seyed Mojtaba Aghaie, Davoud Sadeghi, Hossein Samiei-Abianeh, Alireza Felegary

**Affiliations:** Department of Biology, Faculty of Basic Sciences, Imam Hossein Comprehensive University, Tehran, Iran

**Keywords:** Salmonella Typhimurium, SipD protein, LptD protein, Immunoglobulin Y, Nanoparticle, Alginate

## Abstract

**Introduction:**

*Salmonella*, a zoonotic pathogen causing gastroenteritis, lacks a preventive vaccine. Passive immunization with IgY antibodies derived from immunized chickens has shown potential for treating bacterial infections. This study investigated the therapeutic efficacy of entrapped IgY targeting recombinant SipD and LptD proteins from *Salmonella* Typhimurium.

**Methods:**

The recombinant protein was expressed in *E. coli* BL21 (DE3) and purified using Ni-NTA affinity chromatography. Hens were immunized with the purified protein, and the resulting IgY was entrapped into alginate nanoparticles. The shape of spherical nanoparticles and their size in the nanometer range were determined by SEM and DLS analysis. The therapeutic efficacy of free and alginate-entrapped IgY against *S.* Typhimurium was evaluated in mice at 1, 50, and 100 LD_50_ bacterial doses.

**Results:**

The purified IgY concentration in each egg yolk was 6 mg/ml (35 mg/egg). Physicochemical and structural characterization revealed spherical nanoparticles with a diameter of 157.1 nm and a negatively charged surface (zeta potential of −35.6 mV). The loading efficiency of IgY into alginate nanoparticles was 95.5 %. In a challenge test with 100 LD_50_ of *S*. Typhimurium, all mice receiving alginate-entrapped IgY survived, whereas half of the mice receiving non-entrapped IgY died within 7 days.

**Conclusion:**

Our results indicate that IgY antibodies entrapped in alginate nanoparticles may offer therapeutic effect against *S*. Typhimurium infection.

## 1 Introduction

Infectious gastroenteritis is one of the most important and leading causes of morbidity and mortality, affecting children and adults worldwide [1]. A variety of intestinal pathogens, including *Salmonella* species, are transmitted through contaminated food or water, leading to gastroenteritis infections [2]. *Salmonella* is the most common cause of these infections [1].*Salmonella* is a genus of gram-negative bacteria. Among *Salmonella* species, *enterica* species is responsible for causing salmonellosis in humans.

The World Health Organization (WHO) reports that *Salmonella* is a major contributor to global morbidity and mortality. Typhoid fever, caused by *Salmonella*, affects 17 million people annually, resulting in 600,000 deaths. Moreover, non-typhoidal *Salmonella* infections lead to 1.7 billion cases and 3 million deaths each year [3]. *Salmonella* Typhimurium, a strain of non-typhoidal *Salmonella*, is commonly transmitted through contaminated food or water [4,5], leading to outbreaks of foodborne illness [6]. This bacterium exhibits a wide geographical and host range, making it a significant public health concern [7].

*Salmonella* Typhimurium is a foodborne pathogen that can cause a range of illnesses, from mild gastrointestinal symptoms to severe systemic infections. After entering the body through contaminated food or water, the bacterium invades the small intestine [8], leading to inflammation and diarrhea. In some cases, *S*. Typhimurium can spread to the bloodstream, causing bacteremia and complications such as pneumonia and meningitis [9].

One of the promising approaches to prevent *S.* Typhimurium infection is to inhibit the formation of LPS (lipopolysaccharide) and its attachment to host cells. The transfer of LPS to the outer membrane requires different transport proteins, among which LptD carrier proteins play an essential role. These carrier proteins facilitate the transfer of LPS out of the outer membrane, ultimately responsible for its final transfer to the outside of the cell [10,11]. A subset of *Salmonella* effector proteins facilitates the translocation of bacterial effector proteins across the host cell membrane. These transporters form a host cell membrane channel, allowing bacterial agents to pass through. These transporters are required for functional pore formation during *Salmonella* infection and host cell entry. Among these proteins involved in the type 3 secretion system, the SipD protein has a final and significant role that was targeted. It has also been shown that these proteins play an essential role in the intimate relationship between *Salmonella* and mammalian cells [12].

The increasing prevalence of antibiotic-resistant bacteria poses a significant global health threat. While antimicrobial resistance is a natural phenomenon, the misuse of antibiotics is a major contributor to its spread. Consequently, novel strategies are needed to combat AMR. Passive immunization using egg yolk-derived IgY antibodies (IgY) administered orally, nasally, or topically has shown promise in treating bacterial infections in both animals and humans [13].

IgY technology enables large-scale and low-cost Production of antibodies for passive immunotherapy and prophylaxis. This technology has been successfully tested against various bacterial and viral infections. IgY also exhibits strong binding affinity to target antigens and can be quickly produced against conserved mammalian proteins due to the evolutionary divergence between birds and mammals [14,15]. IgY is a promising candidate for preventing gastrointestinal infections due to its unique properties. Unlike mammalian antibodies, IgY does not react with rheumatoid factors, which can be beneficial in individuals with autoimmune conditions [16,17]. Additionally, IgY inhibits the activation of the complement system, reducing the risk of adverse reactions and inflammation. These factors, combined with its potential for large-scale production, make IgY an attractive option for combating infectious diseases [[18], [19], [20]].

Alginate or alginic acid is a natural polysaccharide polymer composed of mannuronic acid and glucuronic acid, which are linearly connected. It is primarily extracted from seaweed, with a molecular weight ranging from 32 to 400 kg/mol [21]. Due to its anionic nature, alginate links polymers when combined with calcium chloride (CaCl_2_), wherein calcium has a positive charge, allowing for protein loading into the polymer matrix [22]. Alginate nanoparticles demonstrate swelling behavior in response to significant pH changes (e.g., during passage through the stomach and entry into the intestine), making them ideal carriers for gastrointestinal drug delivery [23].

Many studies have demonstrated the efficacy of IgY antibodies in preventing various bacterial infections. For instance, Hadi et al. generated IgY antibodies against a recombinant chimera protein containing key immunogens from *Shigella*, demonstrating their effectiveness in neutralizing *Shigella* toxins and bacteria [24]. In another study, Bakshi et al. successfully encapsulated IgY antibodies against *E. coli* O157:H7 in sodium alginate nanoparticles, enabling their safe passage through the gastrointestinal tract and prophylactic protection against infection [23].

The main goal of this study is to produce IgY against the recombinant chimeric protein containing two immunogens, SipD and LptD, from *S*. Typhimurium, which is called rLPSI. Subsequently, we aim is to investigate the therapeutic effects of IgY, in the forms of Non-entrapped IgY and Alginate-entrapped IgY.

## 2 Material and methods

### 2.1 Expression and purification of recombinant protein

In the previous study, a chimeric gene containing SipD and LptD was designed, synthesized, and cloned into the pET28a vector [2]. The recombinant pET28a-*rLPSI* plasmid was then transferred into *E*. *coli* BL21 (DE3) bacteria using the heat shock method. The transformed bacteria were the cultured in a medium supplemented with kanamycin (80 μg/ml) to select cells that had successfully incorporated the plasmid.

To induce protein expression, β-D-1-isopropyl thiogalactoside (IPTG) was added to the culture medium. This triggered the expression of rLPSI from the inserted gene. To optimize expression conditions, the culture was incubated at different time points (4 and 5 h), concentrations of IPTG (0.5 mM and 1 mM) and temperatures (25 °C and 37 °C). These variations were assessed to determine the optimal conditions for achieving maximum protein yield.

The cell pellets were resuspended in lysis buffer (100 mM NaH2PO4, 10 mM Tris-HCl, 8 M urea) and sonicated 5 times for 30 s at 1 min intervals. After centrifugation, the supernatant was loaded onto a Ni-NTA column. The column was washed with 5 mL and 2 mL of wash buffer containing 8 M urea with pH 6.3 and pH 5.9, respectively. The recombinant protein was eluted with PBS containing 250 mM imidazole. The urea and imidazole were removed by stepwise dialysis, gradually reducing the concentration of these reagents in the dialysis buffer over several steps.

Finally, the purified protein was analyzed using 12 % SDS-PAGE electrophoresis, and its purity was determined based on the relative band intensity using GelAnalyzer 19.1 software. The protein concentration was measured using the Bradford assay.

### 2.2 Immunization of chickens by rLPSI

25-week-old Shaver line laying hens obtained from the Imam Hossein University Animal House were divided into test and control groups. The test group was immunized subcutaneously with 100 μg of rLPSI recombinant protein, followed by three booster injections at 2-week intervals.

Montanide ISA 70 VG (Seppic, Paris, France), a highly effective and safe adjuvant, was used for administration to the animals. The antigenic solutions were prepared in 300 μl volumes and mixed thoroughly with 700 μl of Montanide ISA 70 VG adjuvant for a total injection volume of 1 mL per dose/hen. For control chickens, 300 μl of PBS was mixed with 700 μl of adjuvant. All animals were maintained in clean, standard conditions at the Imam Hossein University Animal Care Facility, adhering to the Care and Use of Laboratory Animals guidelines.

Blood samples were collected from the chickens one week after each injection. The serum was separated and stored at −20^o^c for antibody titration analysis using the ELISA method.

Antibody titers were determined using an enzyme-linked immunosorbent assay (ELISA). Maxisorp plates (Nunc) were coated with 5 μg of rLPSI in a carbonate-bicarbonate buffer (0.05 M, pH 9.6) and incubated overnight at 4 °C. The wells were washed three times with PBST buffer (Phosphate-Buffered Saline with 0.05 % Tween 20) and blocked with 5 % skim milk in PBST for 60 min at 37 °C. Washed plates were incubated with serially diluted serum (1:200 to 1:25600) and IgY (20000 ng–19 ng) samples for 60 min at 37 °C. After washing, 100 μl of Goat anti-chicken IgG HRP-conjugated antibody (1:10000, Sigma-Aldrich) was added to the wells and incubated for 60 min at 37 °C. Finally, 100 μl of substrate solution containing 60 μg of OPD (o-phenylenediamine) was added to each well plate. The reaction was stopped with 2.5 M H_2_SO_4_, and the absorbance was read at A492 nm on a microplate reader.

### 2.3 Extraction and purification of IgY

One-week post-immunization, eggs were collected and IgY was extracted from the egg yolks using a polyethylene glycol (PEG6000) precipitation method. The eggshells were cracked, and the egg yolks were gently separated from the egg whites. The yolks were then transferred to Falcon tubes and mixed with an equal volume of sterile PBS buffer.

3.5 % (w/v) PEG6000 was added to the sample and stirred gently for 10 min at room temperature. After centrifugation at 10,000 rpm for 10 min at 4 °C, the supernatant was removed and filtered. 8 % (w/v) PEG6000 was added, and the sample was stirred for 10 min at room temperature. Following centrifugation, the supernatant was discarded, and the precipitate was dissolved in 10 mL of sterile PBS buffer. This process was repeated with 12 % (w/v) PEG6000, and the final precipitate was dissolved in 1 mL of sterile PBS buffer. After overnight dialysis against PBS buffer, the sample was stored at −20 °C. The purity of the extracted antibody was assessed using 9 % SDS-PAGE and GelAnalyzer 19.1 software.

### 2.4 Preparation of IgY-nanoparticle

Sodium alginate NPs were prepared using the ionic gelation method [23]. Alginate stock solution with a 2 mg/ml concentration was stirred overnight on a stirrer. The solution was then filtered using a 0.45 μm filter to remove any impurities. To load IgY into NPs, 2 mg of IgY was added dropwise to 2 ml of alginate stock solution for 10 min on a stirrer. Then, 1.5 ml of CaCl_2_ solution (1 mg/ml) was added drop by drop to the mixture. The solution was stirred for 45 min at a speed of 1700 rpm at 4 °C to enable proper incorporation of protein into nanoparticles. Then, the solution was centrifuged at 12,000 rpm and 4 °C for 20 min. This step was done to separate nanoparticles from the supernatant solution. The supernatant was collected for further analysis, and the resulting alginate gel was lyophilized and stored at 4 °C.

### 2.5 Investigation of the characterization of alginate-entrapped IgY

To investigate the formation of alginate nanoparticles and the amount of antibody retention, three parameters were calculated: particle yield [22], entrapment efficiency [23], and loading capacity [23] (Table 2). Three different concentrations of antibodies (0.5, 1, and 2 mg) were entrapped in alginate nanoparticles. The alginate gel was lyophilized, and the amount of free IgY in the supernatant was measured.

The size distribution and zeta potential of the nanoparticles were analyzed using Dynamic Light Scattering (DLS), comparing IgY-entrapped nanoparticles to empty nanoparticles as a control. The morphology of the nanoparticles was also examined using Field Emission Scanning Electron Microscopy (FE-SEM).

The FE-SEM images were captured at a magnification of 20,000 times (20KX), allowing for a detailed visualization of the surface and structural characteristics of the nanoparticles. Finally, the cytotoxicity of non-entrapped IgY and Alginate-entrapped IgY was assessed *in vitro* using the MTT assay test. The test was performed to investigate the potential cytotoxic effects of non-entrapped IgY and Alginate-entrapped IgY on the target cells.

### 2.6 Investigating the *in vitro* release behavior of alginate-entrapped IgY

To assess the stability of alginate-entrapped IgY, lyophilized samples were incubated in a specific buffer (NaHCO3 0.1 M, Sodium Citrate, pH = 8) to release the IgY. The release process was conducted for 1 h at 37 °C and 150 rpm. The released IgY was then subjected to electrophoresis using a 9 % SDS-PAGE gel and a control sample of non-entrapped IgY mixed with sample buffer, both with and without mercaptoethanol.

The activity of the released IgY was evaluated using an indirect ELISA and compared to that of non-entrapped IgY. To simulate IgY release in gastrointestinal conditions, two groups of samples (each with 3 repetitions) were incubated in stomach-simulating buffer (SGF; 0.03 M NaCl without pepsin, pH 1.2) and intestinal simulating buffer (SIF; 0.05 M KH_2_PO_4_ without pancreatin, pH 6.8) at different time points (0.5, 1, 2, 4, 6, 8, 12, and 24 h). The samples were incubated at 37 °C and 100 rpm. After each time interval, the samples were centrifuged at 12,000 rpm for 20 min, and the supernatant was replaced with fresh buffer. The concentration of IgY in the supernatant solution was measured using the Bradford method.

### 2.7 Efficacy of IgY against *S*. Typhimurium

To evaluate the efficacy of IgY against *S*. Typhimurium, an oral challenge was performed in mice. Mice were randomly divided into 3 groups and orally challenged with 1, 50, and 100 LD_50_ of S. Typhimurium bacteria, respectively (LD_50_ values were determined in a previous study [2]). Immediately after infection, IgY was administered to each group as shown in Table 1. The mortality rate of the mice was monitored for 14 days to assess the effectiveness of the preventive treatment. Two control groups received only PBS or non-entrapped alginate.

**Table 1:** Summary of mice groups and treatments. Groups, IgY doses, amount of administration of Non-entrapped IgY and Alginate-entrapped IgY and Administration Route.

IgY formulation	IgY doses	Administration Route	Mice	Group name
Non-entrapped IgY	100 μg	Oral (Immediately after infection)		Free IgY (100)
			8^a^	
	200 μg			Free IgY (200)
Alginate-entrapped IgY	100 μg			IgY-NPs (100)
	200 μg			IgY-NPs (200)
Alginate without IgY	–			Alginate
PBS	–			PBS

a Total number of mice: 144 (8 mice per group).

**Table 2:** IgY entrapment efficacy in alginate-entrapped IgY.

**IgY concentration**	**Vol of alginate solution (0.2%w/v)**	**Particle yield (%)**	**Entrapment Efficacy (%)**	**Loading Capacity (mg/g)**
0.5 mg	1 ml	80	96.4	150.6
1 mg	1 ml	95.3	95.5	238
2 mg	1 ml	76.3	58.75	279

### 2.8 Statistic analysis

To determine the results' significance, we performed one-way variance (ANOVA) and *t*-test with a significance level of P < 0.05. The survival rate in the challenge test was checked with the Kaplan-Meier graph using GraphPad Prism 9 software.

## 3 Results

### 3.1 Expression and purification of recombinant LPSI protein

**Fig. 1:**
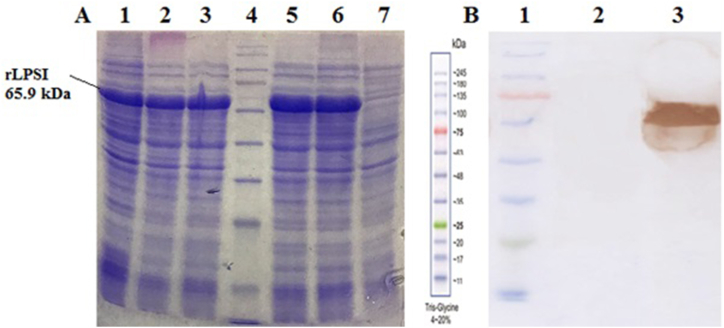
Recombinant protein expression and its evaluation by Western blot method. (A) Optimization of rLPSI protein expression conditions: expression induced by 0.5 mM IPTG in 25 °C (Lane 1), expression induced by 0.5 mM and 1 mM IPTG in 37 °C for 4 h (Lane 2 and 3), expression induced by 0.5 mM and 1 mM IPTG in 37 °C for 5 h (Lane 5 and 6), Non-induced bacteria (Lane 7), *Prestained protein ladder*-SL7011 (Lane 4). (B) Western blot analysis of recombinant protein using anti-His tag antibody: rLPSI protein (Lane 3), BSA protein used as a negative control (Lane 2), *Prestained protein ladder*-SL7011 (Lane 1).

The recombinant protein was successfully expressed at 37 °C for 5 h using 0.5 mM IPTG as an inducer (Fig. 1A). Western blotting with an anti-histidine tag antibody confirmed the identity of the recombinant LPSI protein, which had an expected molecular weight of 65.9 kDa (Fig. 1B).

**Fig. 2:**
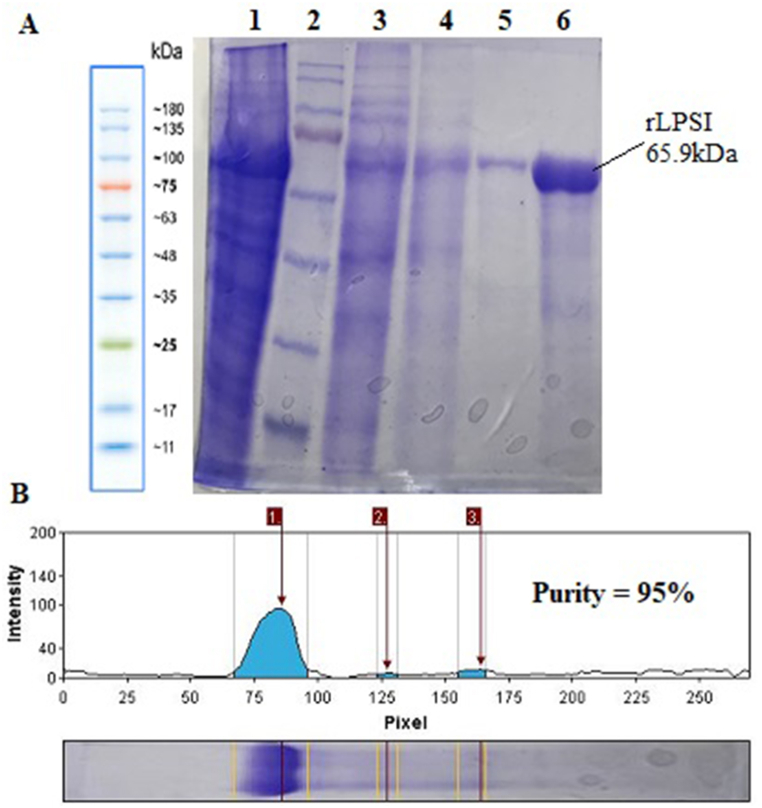
Purification of rLPSI under denatured conditions using a Ni-NTA chromatography column: (A) sample before purification (Lane1), *Prestained protein ladder*-SL7011 (Lane 2), flow-through (Lane 3), washing sample with buffer contain 8m urea with pH = 6.3 and pH = 5.9 (Lane 4 and 5), elution in buffer contain 250 mM imidazole (Lane 6); (B) Software analysis of percent purity of isolated rLPSI.

The recombinant protein was successfully purified using a Ni-NTA column under denatured conditions. SDS-PAGE analysis revealed a single, distinct band corresponding to the recombinant protein, indicating high purity and the absence of non-specific protein contamination (Fig. 2).

### 3.2 Determination of serum IgG titer

**Fig. 3:**
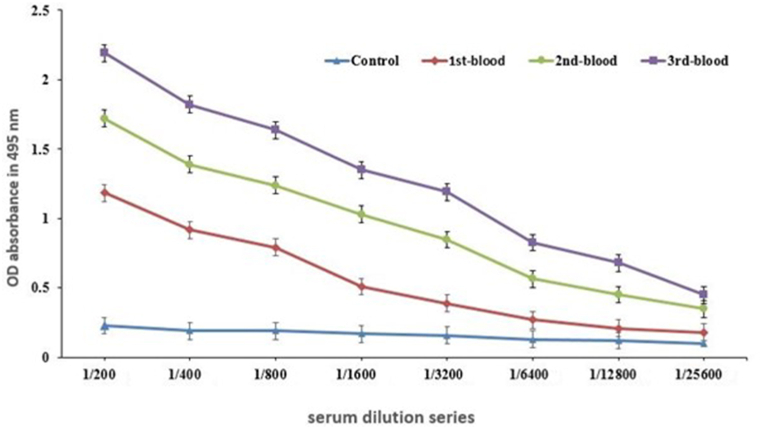
Analysis of antibody titer against rLPSI in serum using the ELISA method.

Analysis of the antibody titer against rLPSI in serum revealed a significant increase in immunized chickens compared to controls following each injection. The results of the indirect ELISA are depicted in the graphs (Fig. 3).

### 3.3 Production and validation of IgY against rLPSI

**Fig. 4:**
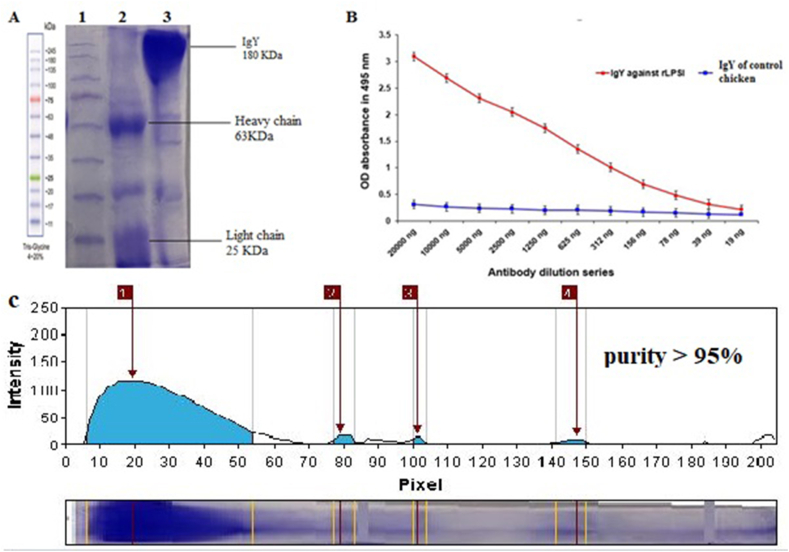
Assessment of IgY Purity and Molecular Weight (A) SDS-PAGE analysis of IgY purification**:***Prestained protein ladder*-SL7011 (Lane 1), IgY with sample buffer containing 2-mercapto ethanol(2-me) (Lane2), IgY with sample buffer without 2-me (Lane3). (B) Analysis of purified IgY antibody titer against rLPSI using the ELISA method. (C) Evaluating IgY Purity with GelAnalyzer 19.1 software.

Following successful purification of IgY from egg yolk, 9 % SDS-PAGE gel analysis revealed distinct bands corresponding to IgY and its light and heavy chains, confirming the expected protein structure (Fig. 4A). ELISA analysis demonstrated the high specificity of the purified IgY towards the recombinant LPSI protein, with reactivity observed at various concentrations (Fig. 4B). Software analysis confirmed a purity of over 90 % for the purified IgY (Fig. 4C).

### 3.4 Preparation and analysis of alginate-entrapped IgY

**Fig. 5:**
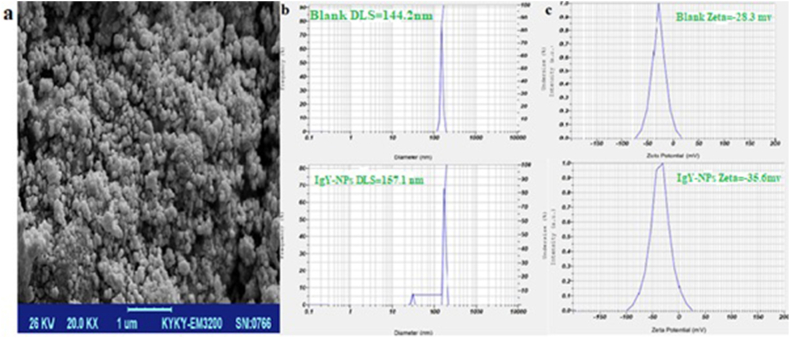
Characterization of morphology, size and zeta potential of alginate nanoparticles. SEM analysis of the surface of Alginate-entrapped IgY showed a spherical shape with smooth surfaces(a). The mean particle size of nanoparticles increased from approximately 144.2 nm–157.1 nm after loading (b), while the zeta potential decreased from −28.3 mV to −35.6 mV(c).

The highest entrapment efficiency for IgY in alginate nanoparticles was achieved with a loading amount of 1 mg/mL (Table 2). Loading IgY decreased the zeta potential of the nanoparticles from −28.3 mV to −35.6 mV (Fig. 5c), while Dynamic Light Scattering (DLS) analysis revealed a slight increase in particle size from 144.2 nm to 157.1 nm (Fig. 5b). Scanning electron microscopy (SEM) images confirmed the expected spherical morphology of the nanoparticles with a nanometer-scale size (Fig. 5a).

**Fig. 6:**
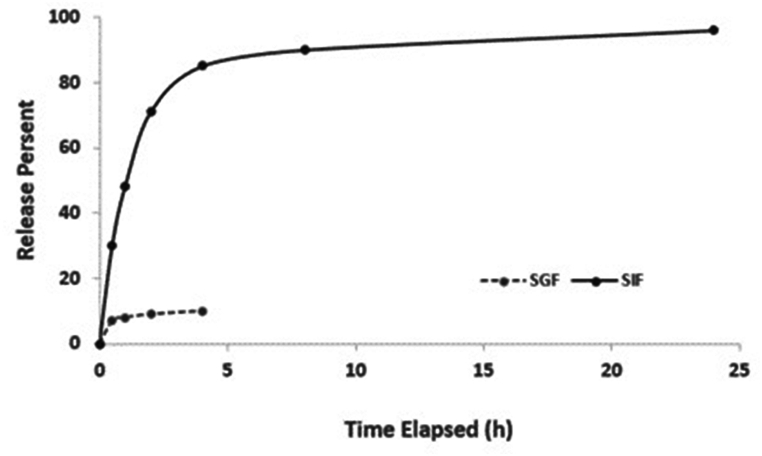
*In vitro* cumulative release of Alginate-entrapped IgY in SGF and SIF buffer. Alginate-entrapped IgY nanoparticles were found to be stable in the stomach environment (SGF) but released the encapsulated IgY completely in the intestinal environment (SIF).

**Fig. 7:**
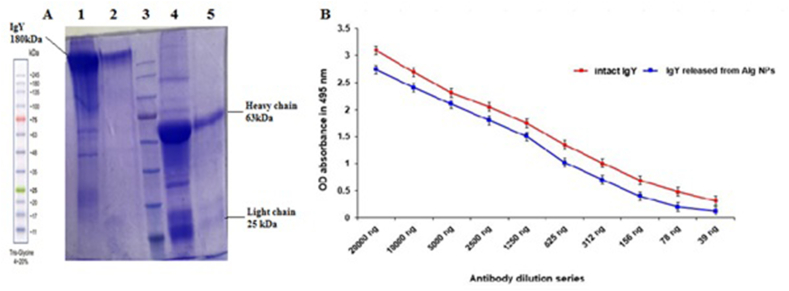
Integrity assessment of IgY released from nanoparticles by SDS PAGE. (A). Intact IgY with sample buffer (Lane 1), released IgY with sample buffer (Lane2), *Prestained protein ladder*-SL7011 (Lane 3), intact IgY with sample buffer containing 2-me (Lane 4), released IgY with sample buffer containing 2-me (Lane 5). (B). Assessing the Immunological Efficacy of IgY Released from Alginate Nanoparticles by ELISA.

To simulate IgY release in gastrointestinal conditions, samples were incubated in simulated gastric fluid (SGF) and simulated intestinal fluid (SIF) for 24 h. While only 10–15 % of IgY was released in SGF, nearly 100 % of IgY was released in SIF (Fig. 6). After release, the integrity of IgY was evaluated using a complete release buffer. SDS-PAGE analysis showed no significant differences between released and non-entrapped IgY, indicating the preservation of molecular structure (Fig. 7A). Additionally, ELISA demonstrated that the binding affinity of released IgY to the antigen remained unchanged, confirming the preserved functionality of alginate-entrapped IgY (Fig. 7B).

The MTT assay examined the cytotoxic effect of non-entrapped IgY and Alginate-entrapped IgY. The test results revealed none of the tested concentrations (0.2, 0.4, 0.6, and 0.8 % w/v) exhibited any toxic effects on the Vero cell line.

### 3.5 Efficacy of IgY against *S*. Typhimurium

**Fig. 8:**
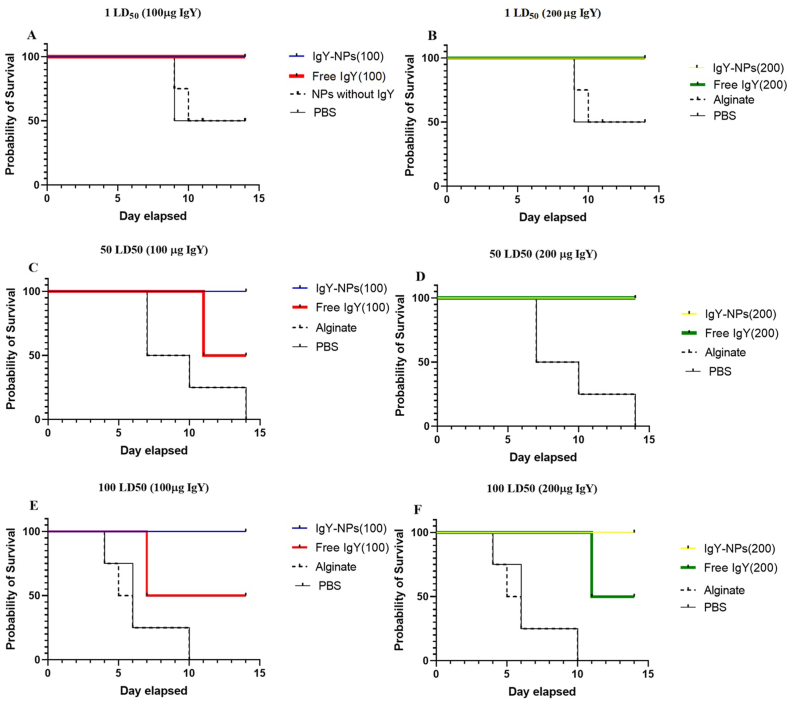
Therapeutic Efficacy of Alginate-entrapped IgY Against *Salmonella* Pathogenicity. The group that received Alginate-entrapped IgY showed the highest survival rate; no deaths were observed, while the survival rate was only 50 % in the Non-entrapped IgY group.

To assess the therapeutic effect of IgY, infected mice were monitored for 14 days. All mice treated with IgY exhibited significant tolerance to *S*. Typhimurium infection compared to controls. Notably, complete survival was observed in all groups receiving alginate-entrapped IgY and in the group receiving non-entrapped IgY at a dose of 1 LD_50_. However, survival rates decreased to 50 % in non-entrapped IgY groups receiving 50 and 100 LD_50_. In contrast, all control mice (Alginate and PBS) receiving 50 and 100 LD_50_ died within 14 days (Fig. 8).

## 4 Discussion

Intestinal pathogens like *Salmonella* pose a significant health risk, especially for individuals with weakened immune systems. *Salmonella* can cause severe digestive problems in healthy individuals and chronic infections in those with compromised immunity [2,25].According to the CDC, *Salmonella* is more pathogenic than previously believed. It can lead to bloody diarrhea, fever, and abdominal cramps, often requiring hospitalization for individuals with severe cases. Children, the elderly, and those with underlying health conditions are particularly susceptible to *Salmonella* infection. *Salmonella* is highly prevalent, but underreporting is common. For every reported case, it's estimated that approximately 30 cases go unreported, making it a serious global health concern [26].

The primary prevention method for *S*. Typhimurium has historically been attenuated and killed vaccines. However, the effectiveness of these vaccines has waned over time [27]. To address this, newer-generation vaccines and antibody therapies (Prophylaxis) offer promising alternatives. Prophylaxis, which involves neutralizing the pathogenic agent, is a critical strategy. Polyclonal and monoclonal antibodies are widely used in prophylaxis to counteract pathogens [16]. To prevent *S*. Typhimurium infection, passive immunization using chicken IgY antibodies has been explored.

IgY has garnered significant attention as a promising therapeutic option for combating gastrointestinal infections due to its unique properties. Notably, IgY is known for its non-reactivity with rheumatoid agents and its ability to inhibit complement system activation [26,27]. These characteristics reduce the risk of adverse reactions and inflammation, making IgY an attractive therapeutic candidate.One of the most significant advantages of IgY is its potential for large-scale production. Each egg yolk can contain a substantial amount of specific IgY, ranging from 2 to 10 mg [17]. This remarkable production capacity ensures a continuous and abundant supply of antibodies, making IgY a viable and cost-effective option for therapeutic applications.

The non-invasive nature of IgY production, involving the collection of eggs from immunized hens, further enhances its practicality and ethical appeal as a therapeutic agent [18].IgY's remarkable stability, allowing for long-term storage without significant loss of activity, is another key advantage [12]. This characteristic makes it a viable option for distribution and utilization in various healthcare settings, including those with limited resources.Additionally, IgY production costs are significantly lower than other antibody sources, such as mammalian sera [19]. This cost-effectiveness is crucial, particularly in global health contexts where affordable treatment options are essential to combat infectious diseases.

For intestinal pathogens, oral drug administration is the preferred method. However, protein drugs like IgY require protection from the harsh conditions of the digestive system, including the low pH of the stomach and proteolytic enzymes [28,29]. Nanoparticles have emerged as promising carriers for drug delivery in various medical applications, offering advantages such as controlled release, reduced side effects, enhanced efficacy, decreased dosage frequency, and prolonged drug shelf life in the digestive system [28,30]. Alginate nanoparticles are particularly well-suited for delivering IgY to the intestine due to their unique properties. They exhibit minimal release in acidic conditions and complete release in alkaline environments, mirroring the conditions in the digestive tract. The *in vitro* release behavior of alginate nanoparticles (Alg-NPs) in simulated gastric fluid (SGF) and simulated intestinal fluid (SIF) is demonstrated in Fig. 7.

Purified IgY obtained via PEG6000 precipitation showed a concentration of approximately 6 mg/ml (35 mg per egg yolk) with a purity between 85 and 90 %. Comparable extraction rates and purity were reported by Fathi et al. [25], while Sunwoo [31] and Chae [32] reported higher values, the decrease in IgY concentration compared to the study conducted by Sunwoo and Chae could be due to the difference in IgY extraction and purification method.

After preparation the nanoparticles, the average size of the IgY-entrapped particles increased from 144 nm to 157 nm, indicating protein entrapment within the nanoparticles (Fig. 6). Our findings on the size of nanoparticles were different from Moradhaseli et al. [33] and Cook et al. [34], this difference is probably due to changes in the stirring speed during the synthesis of nanoparticles as well as the speed of adding the ionic linker (CaCl_2_). The entrapment of IgY in nanoparticles was optimized with a weight-to-weight ratio of Alg:IgY 2:1 (2 mg of IgY entrapped in 4 mg of Alg). In this scale, we have the best and most suitable yield and also the least protein wastage (Table 2). This result was consistent with other studies, although Mallick et al. [35] reported a different ratio of 3:1.

In the analysis of the release behavior, only about 10 % or less of IgY was released in the gastric simulation buffer, this finding indicates that the alginate nanoparticle loses a minimal amount of entrapped protein in the acidic environment, which is The amount of protein is probably at the level of the nanoparticle and is not related to the efficiency of the nanoparticle. Residual IgY was released in the intestinal simulation buffer, indicating the expected release (Fig. 7). This characteristic and the difference in the release in the acidic environment of the stomach and the alkaline environment of the intestine make this nanoparticle very attractive for the use of effective drugs in the intestine and digestive drugs (such as IgY) due to the proper passage through the stomach and no damage to the protein.

Notably, in the challenge test, the loading process in alginate significantly reduced the required amount of IgY, so that only 100 μg of IgY was sufficient to cause preventive effects in the highest amount of bacteria used (100 LD_50_) (Fig. 8). Further research should be conducted on reducing the amount of IgY loading, which can significantly reduce the amount of IgY consumed and thus, the need for production. Although the challenge test showed that non-entrapped IgY showed much lower effects in preventing infection, this effect was not compensated by increasing concentrations, probably due to the destructive effects of digestive enzymes and the entry of all IgY into the intestine at once. Another group of challenge tests showed the effectiveness of encapsulated IgY, which has no additional effect on mortality with increasing concentration.

Similar findings were reported by Hatemzadeh Esfahani et al. [36]. Given that 100 μg of alginate-entrapped IgY (the minimum amount tested) was sufficient to neutralize 100 LD_50_ of bacteria (the maximum amount tested), it is plausible that even lower doses of IgY could be effective against larger bacterial loads.

Hoseini et al. [37] successfully produced IgY antibodies by intramuscular injection of a recombinant protein containing *V. cholerae* antigens. Oral administration of 100 μg of IgY twice resulted in 60 % and 20 % survival in infant mice infected with 1 LD_50_ and 10 LD_50_ of V. cholerae, respectively. Felegary et al. [38] produced IgY antibodies against *Shigella* antigens. Treatment of mice with 10 mg/kg of IgY led to 80 % survival against 10 LD_50_ of *S*. *dysenteriae*. Additionally, Hadi et al. [39] produced IgY against *Shigella* antigens, and treatment of mice with 1000 and 1500 μg of IgY resulted in 70 % and 30 % survival against 10 LD_50_ and 50 LD_50_ of *S. dysenteriae*. Our findings demonstrate a higher survival rate with a lower dose of IgY compared to the studies cited.

## 5 Conclusion

This study employed a nanocarrier, the natural polymer alginate, to deliver IgY to the intestine. The ionic gelation method, known for its high capacity and efficiency, was selected for nanoparticle preparation. Loading 1 mg of IgY in 1 mL of alginate solution yielded favorable results. The nanoparticles produced were of nanometer size and exhibited uniform charge distribution. The release of IgY from these nanoparticles demonstrated their suitability for delivering and maintaining the integrity of IgY in severe digestive conditions. In the challenge test, groups receiving alginate-entrapped IgY exhibited 100 % survival, unlike the groups receiving non-entrapped IgY. This further supports the efficacy of alginate nanoparticles as a delivery system for IgY in combating intestinal pathogens.

## CRediT authorship contribution statement

**Mojtaba zafarmand-samarin:** Writing – review & editing, Writing – original draft, Validation, Supervision, Software, Methodology, Formal analysis, Data curation. **Shahram Nazarian:** Writing – review & editing, Supervision, Methodology, Conceptualization. **Seyed Mojtaba Aghaie:** Software, Methodology, Formal analysis. **Davoud Sadeghi:** Validation, Methodology, Conceptualization. **Hossein Samiei-Abianeh:** Writing – review & editing, Methodology, Data curation. **Alireza Felegary:** Validation, Methodology, Data curation.

## 6 Ethics declarations

The Institutional Animal Care approved the animal experiment, and all applicable international, national, and/or institutional guidelines for the care and use of animals were followed. This article contains no studies with human participants performed by any authors. This research approved by the Ethics Committee of Imam Hossein comprehensive University (code 1401.943).

## Data availability statement

The authors confirm that the raw data and data supporting the findings of this study are available within the article.

## Declarations funding

This research did not receive any specific grant from funding agencies in the public, commercial, or not-for-profit sectors.

## Declaration of competing interest

The authors declare that they have no known competing financial interests or personal relationships that could have appeared to influence the work reported in this paper.
