# Supplementary material for: Therapeutic efficacy of SipD/LptD-specific IgY entrapped in alginate nanoparticles against Salmonella Typhimurium infection
Source: Heliyon. 2024 Oct 24;10(21):e39650. doi: 10.1016/j.heliyon.2024.e39650 (PMC11550739; doi:10.1016/j.heliyon.2024.e39650)
Supplement: Multimedia component 1 [file mmc1.docx]

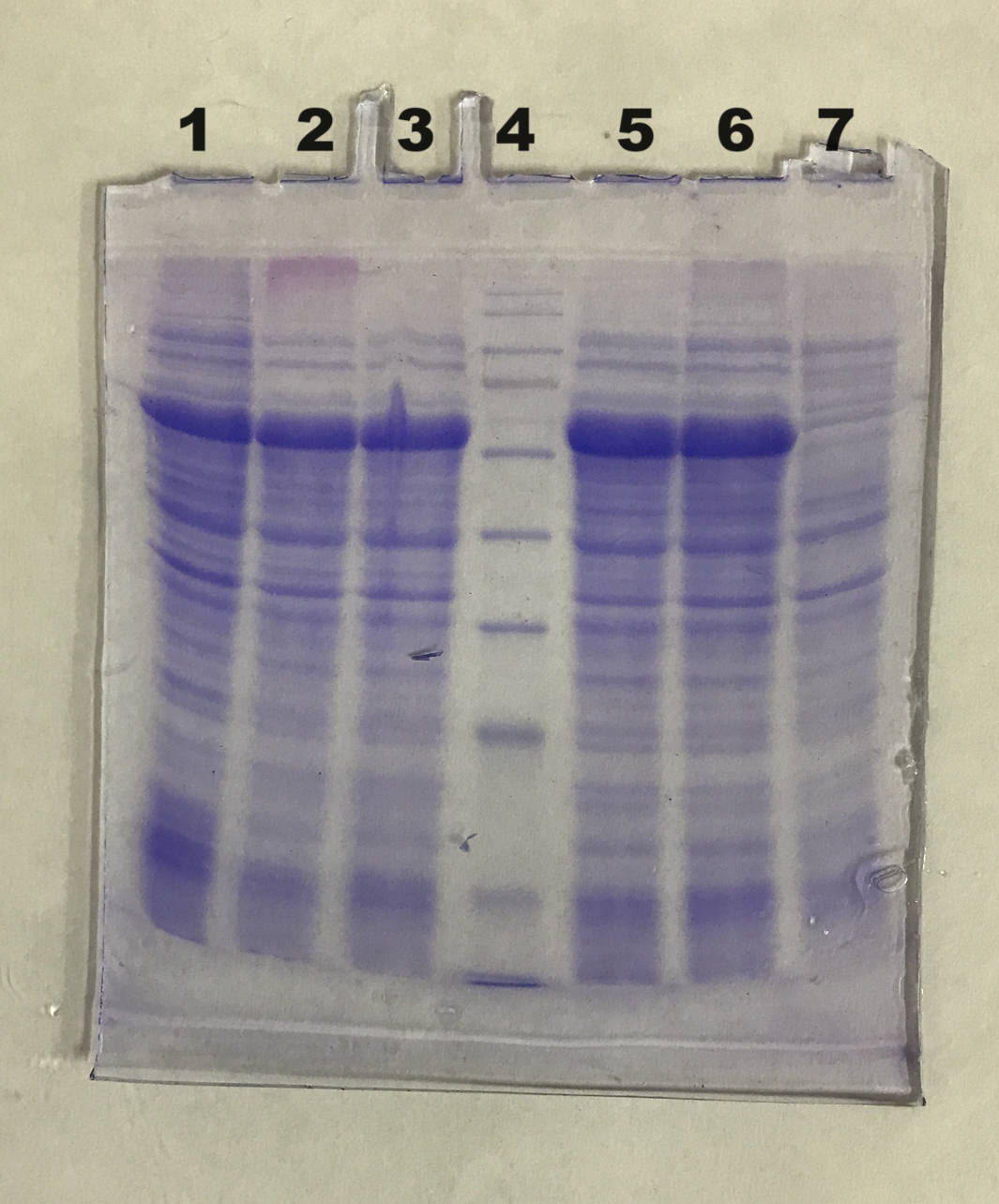


**Fig.1. (A)** Optimization of rLPSI protein expression conditions: expression induced by 0.5 mM IPTG in 25℃ (Lane 1), expression induced by 0.5 mM and 1mM IPTG in 37℃ for 4 h (Lane 2 and 3), expression induced by 0.5 mM and 1 mM IPTG in 37℃ for 5 h (Lane 5 and 6), Non-induced bacteria (Lane 7), Prestained protein ladder-SL7011 (Lane 4).


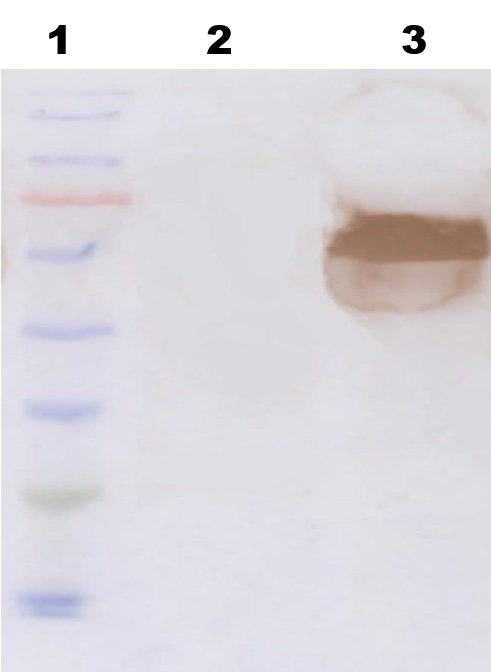


**Fig.1. (B)** Confirmation of recombinant protein using western blotting analysis: rLPSI protein (Lane 3), BSA protein used as a negative control (Lane 2), Prestained protein ladder-SL7011 (Lane 1).


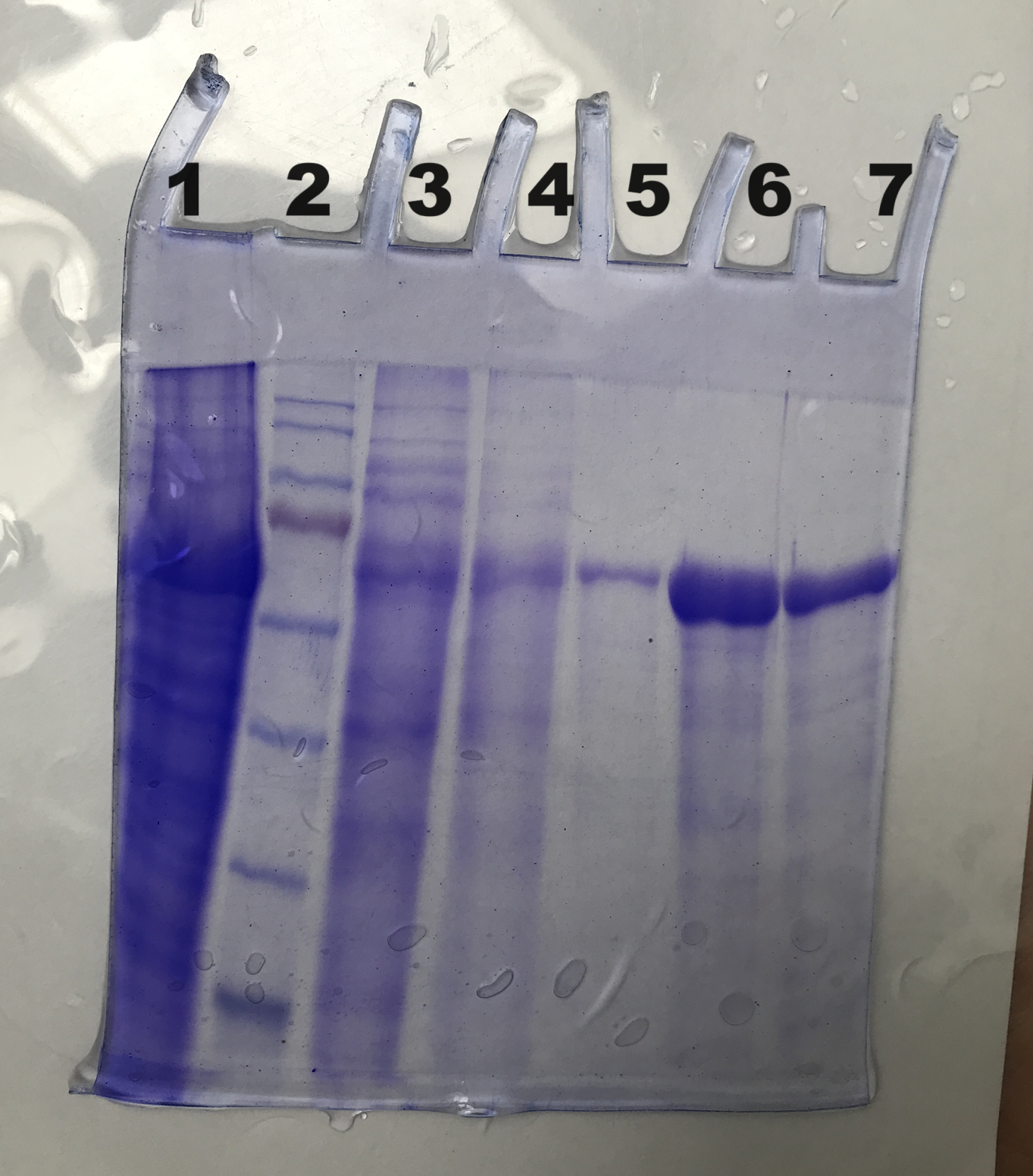


**Fig.2. (A)** Purification of rLPSI under denatured conditions using a Ni-NTA chromatography column: sample before the column (Lane1), *Prestained protein ladder*-SL7011 (Lane 2), flowthrough(Lane 3), washing with pH=6.3 and pH=5.9 from washing buffer (Lane 4 and 5), washing with 250 mM imidazole (Lane 6), washing with 400 mM imidazole (Lane7).


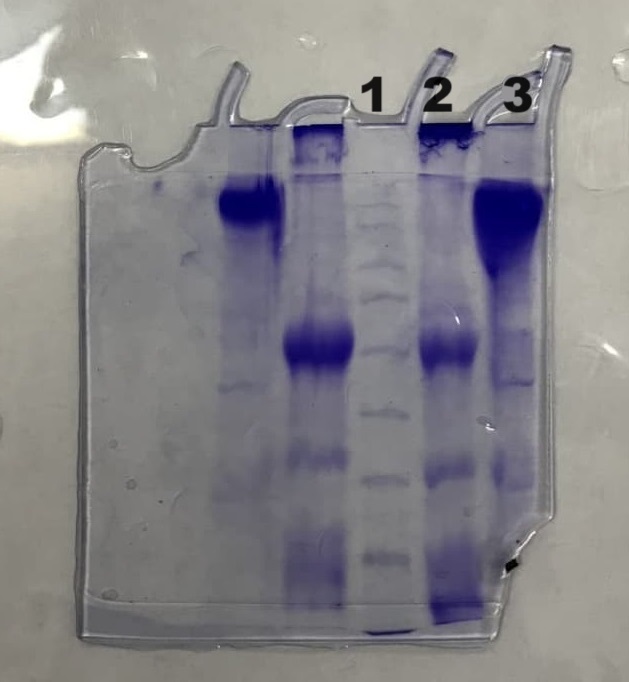


**Fig.4.** (A) SDS-PAGE gel of IgY purification**:** *Prestained protein ladder*-SL7011 (Lane 1), IgY with sample buffer containing 2-mercapto ethanol(2-me) (Lane2), IgY with sample buffer without 2-me (Lane3). Other lanes are related to antibodies with lower concentration, which are not mentioned in this article.


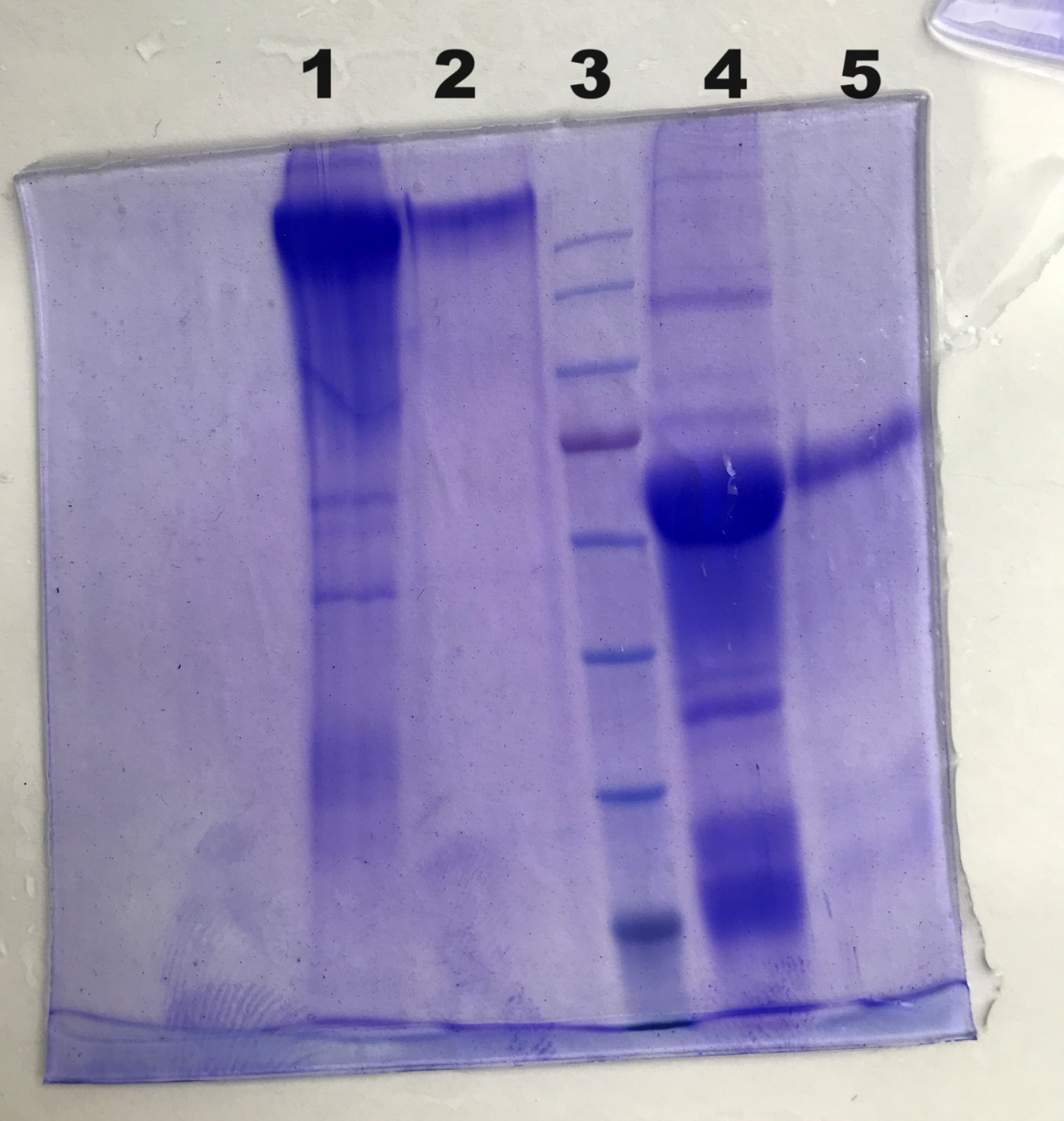


**Fig.7. (A)** Complete release of IgY: intact IgY with sample buffer (Lane 1), released IgY with sample buffer (Lane2), Prestained protein ladder-SL7011 (Lane 3), intact IgY with sample buffer containing 2-me (Lane 4), released IgY with sample buffer containing 2-me (Lane 5).
